# Genome organization and molecular characterization of the three *Formica exsecta* viruses—FeV1, FeV2 and FeV4

**DOI:** 10.7717/peerj.6216

**Published:** 2019-02-20

**Authors:** Kishor Dhaygude, Helena Johansson, Jonna Kulmuni, Liselotte Sundström

**Affiliations:** 1 Organismal and Evolutionary Biology, Faculty of Biological and Environmental Sciences, University of Helsinki, Helsinki, Finland; 2 Department of Animal and Plant Sciences, University of Sheffield, Sheffield, United Kingdom; 3 Tvärminne Zoological Station, Faculty of Biological and Environmental Sciences, University of Helsinki, Hanko, Finland

**Keywords:** RNA virus, *Formica exsecta*, Meta-transcriptome, Organization and characterization, Genome, Comparative analysis

## Abstract

We present the genome organization and molecular characterization of the three *Formica exsecta* viruses, along with ORF predictions, and functional annotation of genes. The *Formica exsecta virus-4* (FeV4; GenBank ID: MF287670) is a newly discovered negative-sense single-stranded RNA virus representing the first identified member of order *Mononegavirales* in ants, whereas the *Formica exsecta virus-1* (FeV1; GenBank ID: KF500001), and the *Formica exsecta virus-2* (FeV2; GenBank ID: KF500002) are positive single-stranded RNA viruses initially identified (but not characterized) in our earlier study. The new virus FeV4 was found by re-analyzing data from a study published earlier. The *Formica exsecta virus-4* genome is 9,866 bp in size, with an overall G + C content of 44.92%, and containing five predicted open reading frames (ORFs). Our bioinformatics analysis indicates that gaps are absent and the ORFs are complete, which based on our comparative genomics analysis suggests that the genomes are complete. Following the characterization, we validate virus infection for FeV1, FeV2 and FeV4 for the first time in field-collected worker ants. Some colonies were infected by multiple viruses, and the viruses were observed to infect all castes, and multiple life stages of workers and queens. Finally, highly similar viruses were expressed in adult workers and queens of six other *Formica* species: *F. fusca*, *F. pressilabris*, *F. pratensis, F. aquilonia, F. truncorum* and *F. cinerea*. This research indicates that viruses can be shared between ant species, but further studies on viral transmission are needed to understand viral infection pathways.

## Introduction

Insect-infecting viruses are known from 16 different virus families, and these show extensive variation in genome, and surface protein structure (Possee & King, 2014). However, to date viruses that infect ants are only known from the *Dicistroviridae* and *Iflaviridae* families, which belong to the order Picornavirales (positive sense single strand RNA viruses). Virus infection by *Dicistroviridae* has been reported in the ant species *Nylanderia pubens* (Nylanderia fulva virus 1 [NfV-1] (Valles et al., 2016), *Linepithema humile* (Linepithema humile virus 1 [LHUV-1]  Lester et al., 2015; Sébastien et al., 2015), *Solenopsis invicta* (Solenopsis invicta virus 1,2,3 [SINV-1, SINV-2 and SINV-3] (Valles, 2012), *Anoplolepis gracilipes* (Black queen cell virus [BQCV] (Cooling et al., 2016). Transmission of these RNA viruses can occur both between closely related and distantly related host-species (Bailey, 1971; Chen & Siede, 2007; Celle et al., 2008; Levitt et al., 2013; Sébastien et al., 2015).

In honey bees (*Apis mellifera*), disease symptoms have been studied at length (Chen & Siede, 2007; McMenamin & Genersch, 2015), and some of those that have been described can be easily diagnosed, like in the case of the deformed wing virus (DWV) (Martin, 2001; Chen & Siede, 2007). In ants, disease symptoms have rarely been described, but cases of severe disease symptoms have been recorded following infection by SINV-3 in *Solenopsis invicta*, which can lead to the death of the colony. In other ant species *Linepithema humile, Solenopsis invicta*) high virus loads has been suggested to negatively impact colony function, such as reduced brood production, decline in foraging efficiency, and death of the colony (Martin, 2001; Chen & Siede, 2007; Sébastien et al., 2015). Gene expression data further suggest that some viruses (e.g., DWV, SINV-1, SINV-3, BQCV) can attack specific developmental stages and castes in eusocial Hymenoptera (Martin, 2001; Chen, Higgins & Feldlaufer, 2005), but the reason for this specialization remains unclear.

The aim of this study was to identify and characterize ant-infecting viruses. Our study species *Formica exsecta* is a widespread native Eurasian ant (Sundström, Chapuisat & Keller, 1996; Sundström, Keller & Chapuisat, 2003). In 2013, we carried out a meta-transcriptomic analysis to search for potential sources of infection in colonies of this ant (Johansson et al., 2013). From these analyses we discovered two viruses, FeV1 and FeV2, with homologies to the *Dicistroviridae* and the *Iflaviridae* families, respectively. These ant viruses have a genome organization similar to some viruses in the *Dicistroviridae* and *Iflaviridae* families that infect honeybees. Here present the primary genome structure and organization of the two viruses reported earlier (FeV1 and FeV2), and present the primary genome structure and organization of a third virus (tentatively named FeV4) discovered using the same data. In addition, we construct a phylogeny at the family level for all three viruses, validate the presence of the viruses from new field collections, and demonstrate the presence of viral RNA of three viruses from different castes and life stages in *F. exsecta*. Finally, we report on the presence and levels of viral RNA of these viruses in the adult worker and queen castes of six other *Formica* species: *F. fusca, F. pressilabris, F. pratensis, F. aquilonia, F. truncorum and F. cinerea.*

## Material and Methods

### Samples

The genomic resources for this study were originally published in Johansson et al. (2013), and Dhaygude et al. (2017) (GenBank Biosample SAMN02046301 –SAMN02046306; Table S1), and comprised samples from colonies of the ant *Formica exsecta* at six localities within a range of 50 km^2^ on the Hanko Peninsula, and the islands outside the Tvärminne zoological station, SW Finland. Screening and annotation of the viruses from these genomic resources was done using RNAseq data published by Johansson et al. (2013) and Dhaygude et al. (2017). The samples for virus screening comprised old reproductive queens (10 queens from 10 colonies, mature individuals), overwintered adult workers (48 workers from 14 colonies, 1–4 workers per colony, mature individuals), newly emerged queens, males, and workers (immature individuals), and pupae, which were differentiated to a stage at which they could be categorized to caste based on external morphology (Dhaygude et al., 2017; Morandin et al., 2015). The pupae were categorized into three developmental stages from each caste: early stage (white cuticle and eyes), intermediate (white cuticle with dark eyes), and late (brown cuticle with dark eyes) (Table S1). To verify the presence of the investigated viruses, an additional new sample of mature workers was collected in July 2013 from 14 colonies (most likely a mix of overwintered and new workers) from the *F. exsecta* population at the Tvärminne zoological station (Sundström, Chapuisat & Keller, 1996; Sundström, Keller & Chapuisat, 2003; Haag-Liautard et al., 2009; Vitikainen, Haag-Liautard & Sundström, 2011) and placed at −80 °C awaiting RNA extraction.

### RNASeq Library preparation and sequencing

Detailed information on RNASeq sample pooling, library preparations and sequencing were previously described in Johansson et al. (2013), and Dhaygude et al. (2017). Briefly, total RNA was extracted from entire individuals using a standard Trizol protocol (TRIsure; Bioline, London, UK), and genomic DNA removed by DNAse I digestion (Fermentas), following the corresponding standard non-strand-specific (NSS) RNASeq protocols of the manufacturers. We generated seven classes of samples: mature overwintered and reproductively active queens, newly emerged queens, queen pupae (all stages pooled), mature overwintered workers, newly emerged workers, worker pupae (all stages pooled), and a mixed pool of newly emerged males and male pupae. We built 14 libraries (2 sets for each sample class; Table S2) using these samples. The two sets of samples were sequenced separately at BGI Shenzhen, China (PE-91 bp), and FIMM Helsinki, Finland (PE-99 bp) to obtain technical, as well as biological replicates for each sample, in both cases using two lanes of Illumina HighSeq 2000. Raw sequencing data for these samples are available on GenBank Biosample (BGI: SAMN02297446 –SAMN02297452 and FIMM: SAMN03799239 –SAMN03799245; Table S2; Dhaygude et al. (2017)).

### Identification of viral sequences

To detect probable exogenous sequences, we used a newly assembled *F. exsecta* transcriptome (Dhaygude et al., 2017), and screened for transcripts that were not of ant origin (Johansson et al., 2013). From this transcriptome, we filtered out the exogenous sequences based on high sequence homology with fungi, bacteria, and viruses, and re-annotated for NCBI taxonomy, using blast best match (GI number to taxonomy). For the detection of whole viral genome sequences, we downloaded all the expressed protein sequences of viruses (DNA and RNA viruses) from the NCBI virus database, and submitted them to an in-house local database. We used the blastx (ncbi-blast-2.2.26 +) program to search this database, with the following parameters: minimum alignment length of 100nt, *E*-value of 0.001, a word size of 11, and a minimum of 30% sequence identity.

In addition to the sequence homology searches for exogenous data, we also carried out functional domain- and protein-related searches to detect additional sequences with properties consistent with viral genomes. Raw reads from the whole transcriptome were aligned back to the detected virus sequences, and the extracted raw reads of virus origin were used to re-construct the genome assemblies of these viruses using Velvet (Zerbino & Birney, 2008; Schulz et al., 2012), and Trinity (Grabherr et al., 2011) *de novo* assemblers. We used two different softwares for the assembly of virus genomes to allow comparison of the results and ensure that the assembled genomes were full-length single contigs, and the absence of gaps.

We further screened all seven transcriptomes available for related *Formica* species (*F. pressilabris, F.fusca, F.cinerea, F. aquilonia, F. truncorum, F.pratensis, and F. exsecta)* (Morandin et al., 2016), to test for the presence of known (FeV1 and FeV2 (Johansson et al., 2013)), and unknown or orthologous viral genomes, specific to each ant species. The RNASeq data were downloaded from the Fourmidable database (Wurm et al., 2009; Morandin et al., 2015; Morandin et al., 2016), and had been obtained from mature workers and queens, collected in the field, and sequenced individually with three biological replicates each, except *F. exsecta* with two biological replicates (Morandin et al., 2016). We then aligned the raw transcriptome data from these seven *Formica* species back to the fully annotated genomes of the *F. exsecta* viruses using the bowtie aligner, and constructed the viral consensus sequences for the ant species that yielded positive matches by parsing BAM alignment files using Samtools (Li et al., 2009).

### Validation of viruses in *F. exsecta*

We validated the presence of the known viruses FeV1 and FeV2, as well as prospective new viruses using RT-qPCR analysis from field-collected workers. We selected two ant control genes GAPDH and RPS9 for RT-qPCR (Table S3); these genes showed minimal variation in relative expression in a previous gene expression study on *F. exsecta* ( Morandin et al., 2014). We first used the original RNA pools, remaining from the transcriptome sequencing to test and optimize primers. Then we pooled 10 individuals from the new field samples collected in July 2013 (in total 140 individuals from 14 colonies), extracted RNA following the Trizol protocol above, and synthesized cDNA using the iscript cDNA synthesis kit (Bio-Rad, Hercules, CA, USA), according to the manufacturer’s protocol. We designed the primers for RT-qPCR virus detection with GeneFisher (Giegerich, Meyer & Schleiermacher, 1996), and Primer-BLAST (Ye et al., 2012), in the highly conserved helicase, RDRP domain, and the highly variable capsid protein. We chose to design primers for several genes, since the diagnostics of rapidly evolving RNA can be challenging, owing to accumulation of mutations that may affect primer sites. We then analyzed individual primers using NetPrimer (http://www.premierbiosoft.com/netprimer/) for primer melting temperature, molecular weight, GC%, and secondary structures. After initial PCR amplification tests, we obtained a single primer pair for each virus, that amplified consistently (Table S3). The primers for all viruses were optimized in the original cDNA pools of RNASeq library with RT-qPCR followed by Sanger sequencing of the PCR product. Sanger sequencing showed that the PCR products exactly matched the targeted sequence obtained from the RNAseq assembly.

For all primers, the RT-qPCR was conducted in 11 µl reaction volumes containing 5 µl iQ Sybr green Supermix (Bio-Rad), 2 µl Forward + Reverse primer mix (5 µM each), 3 µl MQ water, 1µl cDNA (dilution 1:2). The PCR program was 3 min at 95 °C, 5 cycles of 10 s at 95 °C, 30 s at 59 °C, and 30 s at 72 °C, followed by 39 cycles of 10 s at 95 °C, 30 s at 59 °C, and 30 s at 72 °C, and final extension for 10 min at 95 °C. Agarose gel electrophoresis was used to check the expected PCR product size after which the final PCR products were purified using Ampure (Beckman Coulter, Brea, CA, USA) and sequenced using Sanger methods on ABI 3730 DNA analyzer (Applied Biosystems, Foster City, CA, USA).

### Genome organization and molecular characterization

Gene annotation methods for viruses rely on similarity searches, in which known viral genomes are used as templates for annotating novel genomes. These methods successfully cover annotation of most housekeeping genes, but some genes may be missed, either because they are unique to a particular genome, or because they are highly divergent from known homologs. Here we used the GeneMark.hmm Version 2.0 (http://opal.biology.gatech.edu/GeneMark/) software for open reading frame (ORF) prediction, which uses specific models for predicting viral genomes (Besemer, Lomsadze & Borodovsky, 2001). We used heuristic models for gene prediction, in line with software developer suggestions for genomes shorter than 10 kb. The GeneMark results were confirmed with the gene prediction tool FGENESV (http://linux1.softberry.com/berry.phtml??topic=virus&group=%20programs&subgroup=gfin?dv%20) (Besemer, Lomsadze & Borodovsky, 2001), and by visual inspection of both sets of results. Annotation of the predicted gene sets was done using the Blast2Go tool (Conesa et al., 2005) to determine the most likely gene names, gene ontologies, and protein functions. We detected the domains present in proteins using sequence homology search against the viral protein domain database (Pickett et al., 2012).

### Virus phylogenies

To infer virus phylogenies, we obtained all the available full-genome sequences of *Dicistroviridae* (17 genomes), *Iflaviridae* (23 genomes), and the insect infecting *Mononegavirales* (12 genomes) from the GenBank database and added those discovered from the six *Formica* ant transcriptomes (Table S4). Phylogenies for the viruses (FeV1, FeV2) have been described previously (Johansson et al., 2013), and here we complemented the phylogenies with a third virus detected in this study. The phylogenies were constructed separately due to the divergence, and different genome organizations of the viruses. First, the full FeV1 genome sequences, and the partial FeV1-like sequences were aligned to the *Dicistroviridae* family viruses. Second, the corresponding data for FeV2 and FeV2-like sequences were aligned to the *Iflaviridae* family viruses. Finally, the third prospective virus from *F. exsecta,* and the other *Formica* species is new, so we had no prior knowledge of its phylogeny, except that it belongs to the order *Mononegavirales*. We could not achieve reliable alignment at the nucleotide level, probably because the viruses used in the phylogenetic analysis originated from several families, and therefore have a less recent common ancestor. Instead, the phylogeny was constructed at the protein level by exacting and aligning the polymerase protein of 12 viruses in the order *Mononegavirales* (4 *Nyamiviridae* family viruses, 6 *Rhabdoviridae* family viruses, and 1 virus each from the *Paramyxoviridae* and *Bornaviridae* families). Alignments were carried out at the nucleotide level for FeV1 & FeV2 and protein level for FeV4 with the software MAFFT (Katoh et al., 2002), and missing residues were indicated as “N” for all FeV-like short fragments. In addition, we generated consensus sequences using the complete FeV genomes as references. We constructed separate phylogenomic trees for each virus using the Maximum Likelihood program RAxML (Stamatakis, 2014) under a heuristic approach, and the GTR substitution model. The branch supports for the tree topologies were assessed by bootstrap analysis with 1000 pseudoreplicates of the sequences. We repeated the analyses with the short fragments only, to assess the robustness of the phylogenetic tree. The short fragments were derived from similar regions in FeV1, FeV2, and the most closely related *Discistroviridae* and *Iflaviridae* viruses, respectively, similar in length to the FeV1-like, and FeV2-like sequences (325 bp and 375 bp, respectively). A similar comparison was not done for the FeV4-like sequences, as the fragment was much longer, and encompassed almost the entire gene. Although some FeV-like sequences were very short, they nonetheless give an indication of the phylogenetic affiliation of these fragments. Figures were created using FIGTREE version 1.2 (Rambaut, 2012).

### Infection pattern of viruses in *Formica* species

To investigate the occurrence of viral infections across castes, and development in *F. exsecta*, we analysed RNASeq data of virus sequences in the different libraries (pupae, newly emerged queens and workers, old overwintered queens and workers, and males). We aligned all reads from each library to the virus genomes, extracted the aligned regions and counted the reads. To avoid counting misaligned reads, we made sure at least 5 pair-end reads mapped to the virus genomes with >= 25 mapping quality. This read count was used to calculate RPKM values (Reads per kilobase of genome per million reads mapped) for each virus across the different castes and ages. The number of individuals per library varied from 4 to 30. Thus, the RPKM values were adjusted for the number of individuals in each library, and plotted in heat maps. To assess the levels of viral RNA in different castes in closely-related species, we performed the same analyses on the individual queen and worker libraries of *F. pressilabris, F. fusca, F. cinerea, F. aquilonia, F. truncorum, F. pratensis* and *F. exsecta* from the Morandin et al. (2016) study. We tested for significant differences in levels of viral RNA between queens and workers using paired t-tests performed with R (R Development Core Team, 2008).

## Results

In total, we were able to construct three probable full virus genomes from the meta-transcriptomic data of *F. exsecta*. Of these, two (FeV1 and FeV2) were reported earlier (Johansson et al., 2013), but one virus genome escaped detection in that paper owing to low sequence similarity to viral sequences deposited in GenBank, and scant functional information. We henceforth refer to this virus as FeV4. The annotated probable full genomes of the three viruses are available from GenBank ID: kF500001 (FeV1 virus, 9,554 bp), KF500002 (FeV2 virus, 9,160 bp), MF287670 (FeV4, 9,866 bp). The average genome coverage was 6900X for virus FeV1, 3020X for FeV2 and 2651X for FeV4, in the *F. exsecta* transcriptome (Dhaygude et al., 2017). All three viruses were also detected in the new field-collected mature workers; FeV4 was present in nine, FeV1 in two and FeV2 in one out of the 14 colonies sampled. Two colonies were infected with more than one of the investigated viruses, one with FeV1 and FeV4, and one with FeV2 and FeV4 (Table S5).

### FeV1 virus

**Figure 1:**
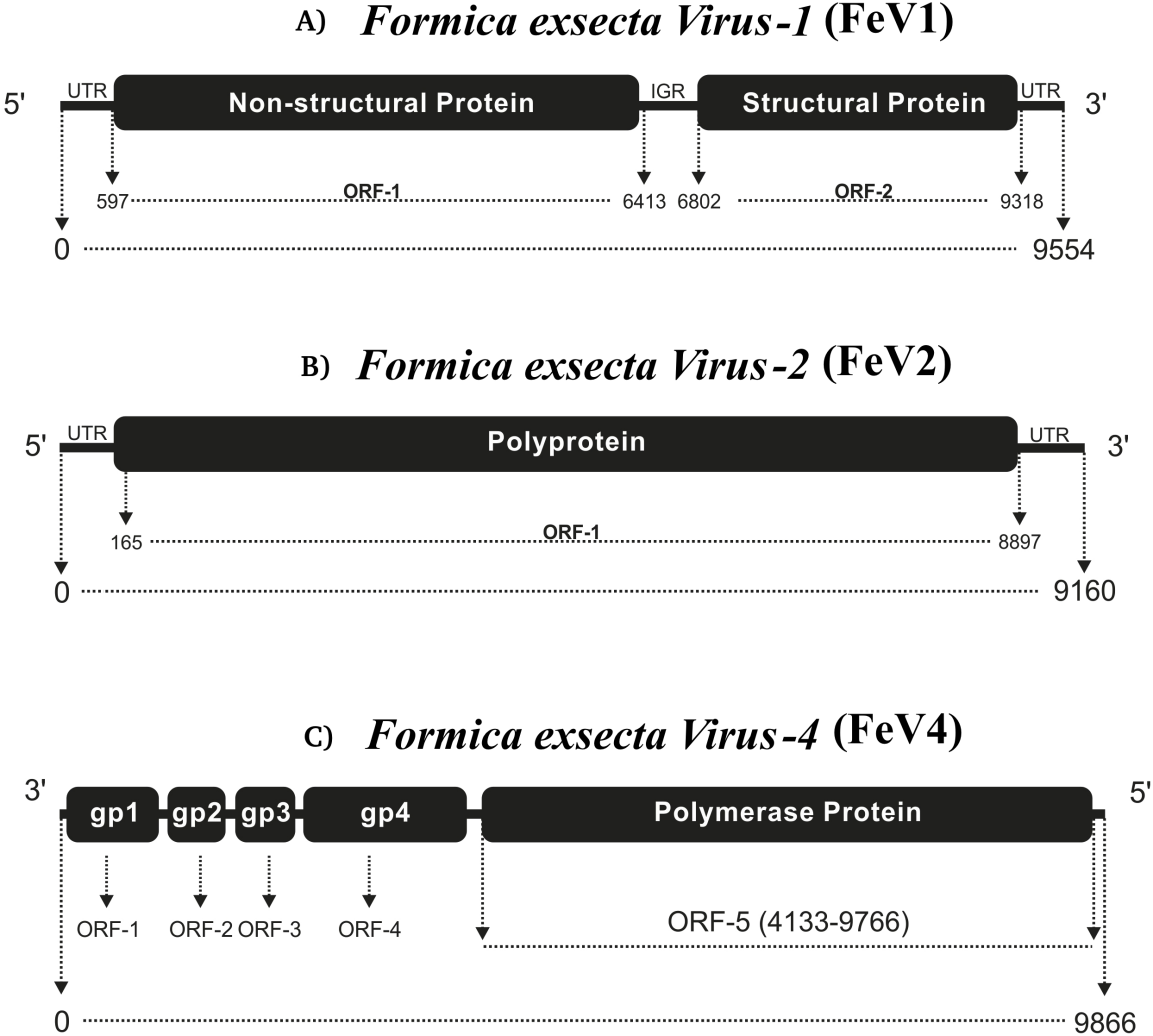
Comparative genome architecture of the three *Formica exsecta* viruses FeV1 (A), FeV2 (B), and FeV4 (C). The figure shows the orientation of the genome, the horizontal black bars indicate the number of cistrons, and the text in them the type of protein. The relative positions of ORFs are indicated by rectangles.

The FeV1 is a linear, positive sense, single stranded RNA virus and it has an A/U rich genome (FeV1 [33.04% A, 28.72% U, 18.52% C, 19.72% G]) (Table 1). Its genome is monopartite dicistronic (Fig. 1A), and contains two open reading frames (ORF): nucleotides 597-6413 (ORF1) and 6802-9318 (ORF2). ORF1 is annotated as a non-structural gene, which encodes a polyprotein of 1938 amino acids, with three sequence motifs for helicase (Hel), 3C-like cysteine proteinase (Pro), and RNA-dependent RNA polymerase (RdRp), in this order. ORF2 encodes a polyprotein of 838 amino acid that originates capsid proteins (Bonning, 2009). Together ORF1 and ORF2 cover almost 87% of the FeV1 genome (Fig. 1A), whereas the remaining 13% of the genome consists of non-coding regions (5′ UTR, 3′ UTR and intergenic regions). Distinct internal ribosome entry sites (IRES) are located in the 5′ UTR and the IGR.

**Table 1:** Genomes of *Formica exsecta* viruses and their characteristics.

Virus	Family/Order	Genome length (bp)	ORFs	Genome region covered by gene (%)	Base composition
FeV1	Dicistroviridae	9,554	ORF1: 597-6413,	87	33.04% A, 28.72% U,
			ORF2: 6802-9318		18.52% C, 19.72% G
FeV2	Iflaviridae	9,160	ORF1: 165-8897	95	32.26% A, 32.09% U,
					14.54% C, 14.54% G
FeV4	Mononegavirales	9,866	ORF1: 2-1171,	96.2	31.16% A, 23.92% U,
			ORF2: 1231-1782,		22.94% C, 21.98% G
			ORF3: 1866-2408,		
			ORF4: 2502-4094,		
			ORF5: 4133-9766		

A blast search to GenBank showed that the FeV1 shows highest similarity to viruses from the *Dicistroviridae* family, with the closest similarity to the Kashmir bee virus (KBV). The FeV1 and KBV are about 75% identical across the entire genome (with 97% genome coverage), with considerable differences between them in the 3′ non-translated region (23% nucleotide identity), 5′ non-translated region (67% nucleotide identity), in the helicase, and 3C-protease domains of the non-structural polyprotein (60% amino acid identity), and in a 110 amino acid stretch of the structural polyprotein (50% amino acid identity).

**Figure 2:**
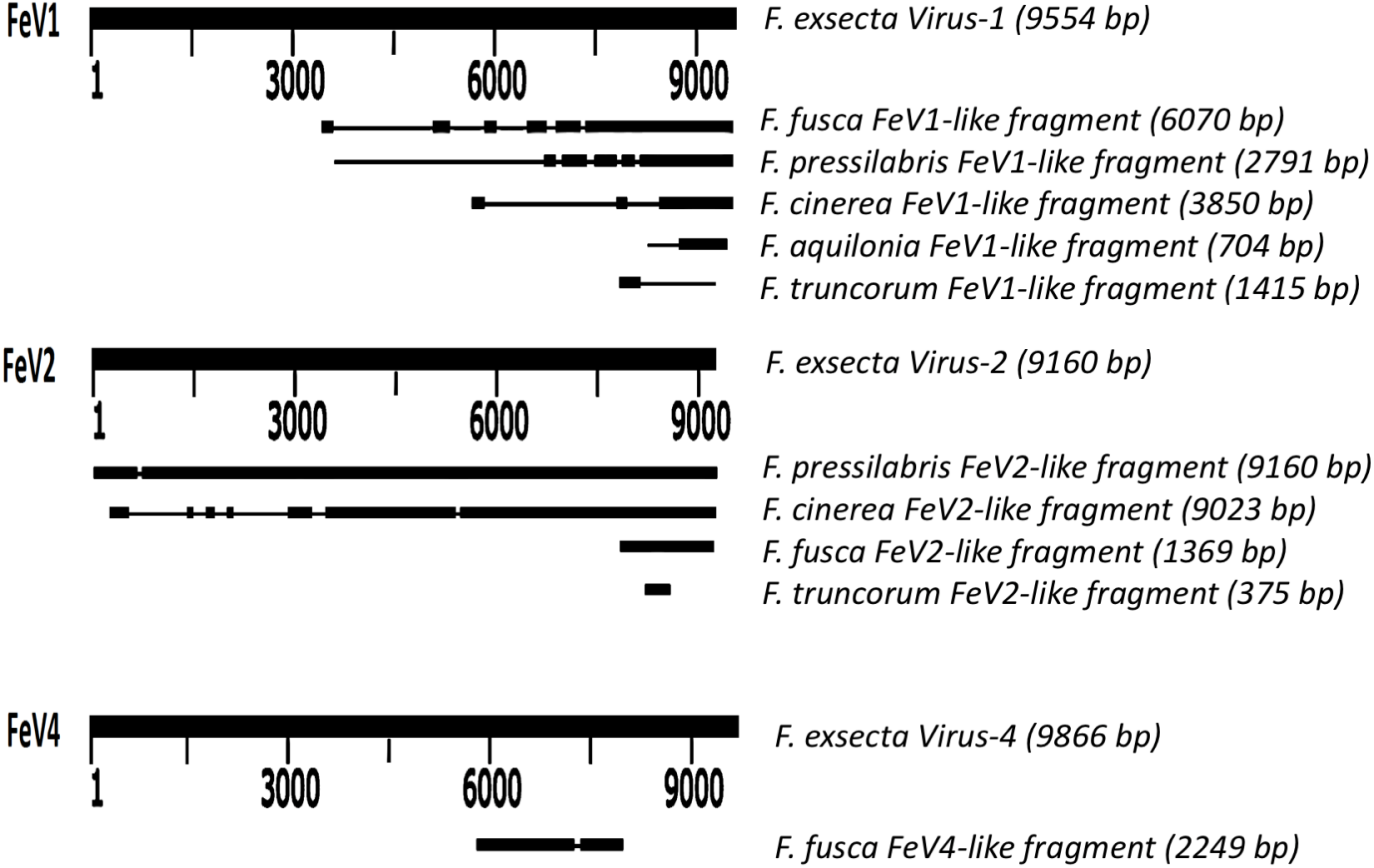
Schematic representation of sequence alignment of *Formica* virus-like fragment.

**Figure 3:**
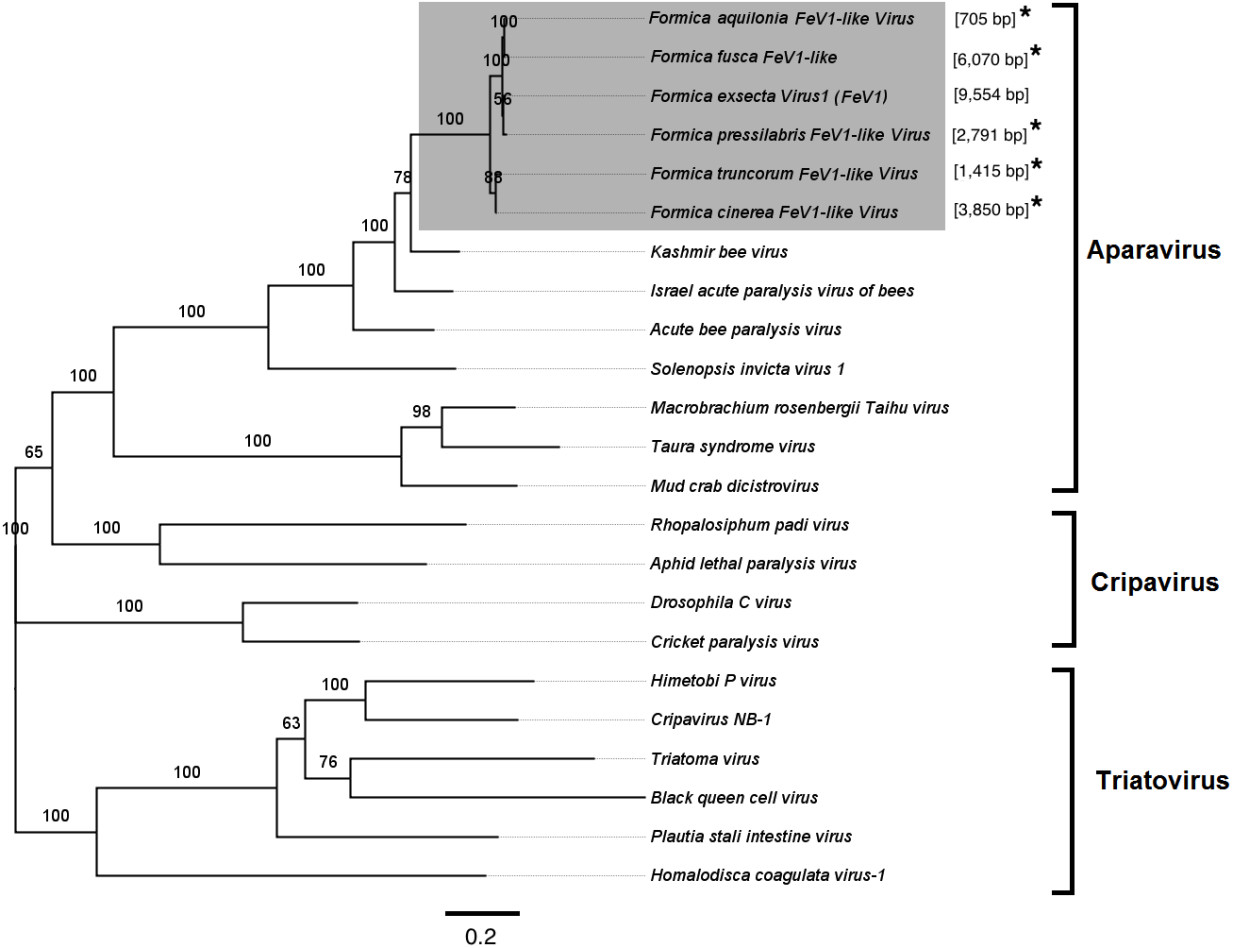
Phylogenetic assignment for FeV1. An unrooted phylogenetic tree was derived from the whole genome sequences of the *Discistroviridae* family viruses, along with the partial or probable full genome of the FeV1-like sequences from different *Formica* host species. The *Formica* virus clade is highlighted in gray and bootstrap values are shown above the branches. The branches between the FeV1-like sequences are preliminary, given that the sequences of these viruses (with ambiguous/uncalled bases) are only partial. An asterisk (*) denotes short FeV1-like sequences.

Screening the RNAseq data of other *Formica* species (mature workers and queens) revealed FeV1-like sequences in *F. pressilabris, F. fusca, F. cinerea, F. aquilonia, F. truncorum*. The sequences were similar over a length of 704 to 6,070 bp, Table 2; Fig. 2). The FeV1 and FeV1-like sequences from these ants showed high sequence similarity (89–98% nucleotide level). The short sequence fragments from the other *Formica* species notwithstanding, the maximum likelihood phylogeny for FeV1 shows 100% bootstrap support, indicating a common ancestor of FeV1 and FeV1-like sequences in *Formica* (Fig. 3; Fig. S1A). The FeV1 clearly clustered with other social insect viruses including the Kashmir bee virus, the Acute bee paralysis virus, and the Israeli acute paralysis virus. Altogether, the phylogenomic tree showed three main clades largely corresponding to three separate virus genera: *Cripavirus*, *Triatovirus* and *Aparavirus*, with the social insect viruses placed in the *Aparavirus* clade (Fig. 3). However, the four *Cripavirus* genera did not cluster together, but two genera clustered with *Aparavirus* and two by themselves. We note that the bootstrap support for this particular branch was only 65%.

**Table 2:** Alignment statistics for FeVs-like sequences (FeV1-like, FeV2-like, FeV4-like), obtained from *F. aquilonia, F. cinerea, F. fusca, F. pressilabris* and *F. truncorum* species (raw data from Morandin et al., 2016), and aligned to the FEX virus genomes obtained from *F. exsecta*.

	FeV1-like					FeV2-like				FeV4-like
	*F. aquilonia*	*F. cinerea*	*F. fusca*	*F. pressilabris*	*F. truncorum*	*F. cinerea*	*F. truncorum*	*F. fusca*	*F. pressilabris*	*F. fusca*
GenBank Accession	MF287660	MF287661	MF287662	MF287663	MF287664	MF287668	MF287665	MF287667	MF287666	MF287669
Length (bp)	704	3850	6070	2791	1415	9023	375	1369	9160	2249
Ambiguous bases (bp)	49	2204	2688	456	931	2684	100	50	120	121
Alignment length (bp)	666	1668	3405	2337	558	6461	320	1347	9040	2159
Percentage Identity	97,00	93,00	93,00	89,00	98,00	91,00	79,00	92,00	91,00	80
Identical bases (bp)	646	1551	3167	2079	547	5879	253	1239	8226	1700
Non-identical bases (bp)	20	117	238	258	11	582	67	108	814	459

**Figure 4:**
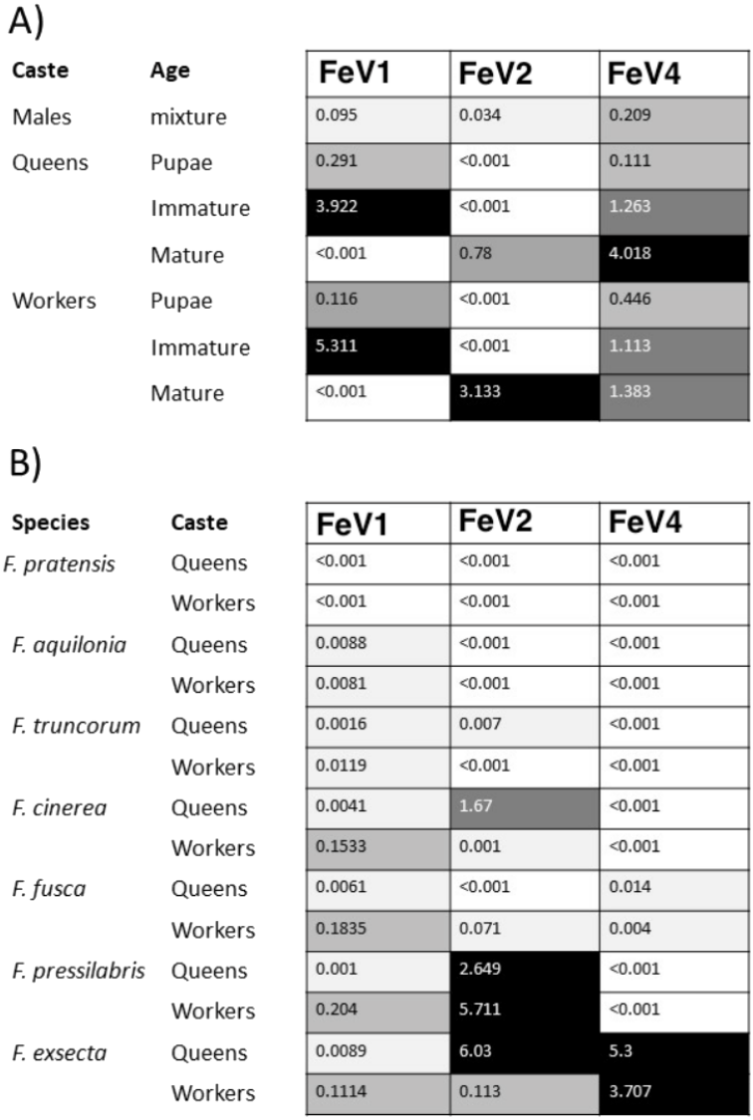
Heat map showing the viral RNA levels of FeV1, FeV2, and FeV4. Heat map showing the viral RNA levels of FeV1, FeV2, and FeV4 in different castes and age classes of *F. exsecta* (A), and in *Formica* species (raw data from Morandin et al., 2016) (B). The heat map is based on transformed (LNx+1) RPKM values (Tables S6, and S7). Values below 0.001 were classified as having no virus RNA.

The FeV1 virus was found in all castes, and in all developmental stages of workers and queens of *F. exsecta* (Fig. 4A). Since male data were pooled (after RNA extraction) we cannot determine virus presence in different developmental stages in males. In females, the viral RNA levels were considerably higher in immature workers and queens than in pupae or in the pooled male samples, whereas the levels were negligible in mature queens and workers (Fig. 4A; Table S6). FeV1-like fragments were found in variable amounts in the other investigated *Formica* species (Figs. 4B; 2; Table S7), with workers of three species (*F. pressilabris, F. cinerea, F. fusca*) showing non-negligible levels of FeV1-like fragments (Fig. 4B; Table S7).

### FeV2 Virus

FeV2 is a linear, positive sense, and single stranded RNA-virus, with nucleotide-level similarity to viruses from the *Iflaviridae* family, and has an A/U rich genome (FeV2 [32.26% A, 32.09% U, 14.54% C, 14.54% G] (Table 1). The genome is monopartite with only one ORF that starts from 165 bp and ends at 8,897 bp, covering nearly 95% of the genome (Fig. 1B). ORF1 from FeV2 was annotated as a structural gene encoding a polyprotein (2910 amino acids) that contains different catalytic subunits, including RDRP, Helicase, and the picornavirus capsid protein domain. The structural proteins are encoded in the N-terminal part of the ORF1, and the nonstructural ones in the C-terminal part.

FeV2 showed the closest nucleotide similarities with the deformed wing virus (71% identity, 11% coverage), the Kakugo virus (71% identity, 19% coverage), and the *Varroa destructor* virus (67% identity, 13% coverage). The nucleotide level similarity with these viruses was low (only 10–20% genome coverage), but the protein level similarity was high. This suggests that FeV2 also shares genomic architecture with all three viruses, (ORF: 90% coverage and 40% identity).

**Figure 5:**
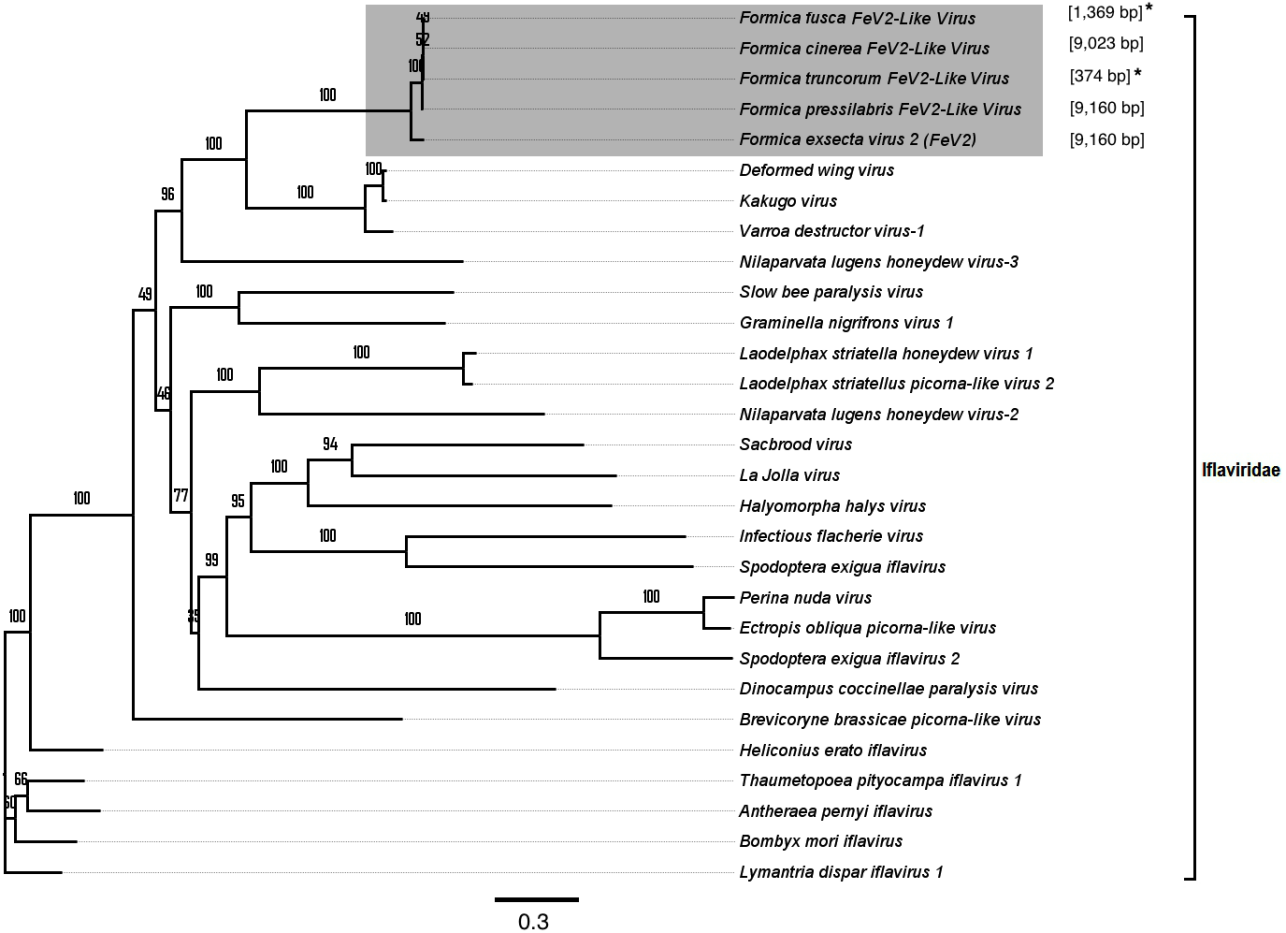
Phylogenetic assignment for FeV2. An unrooted phylogenetic tree was derived from the whole genome sequences of the *Iflaviridae* family viruses, along with the partial or probable full genome of the FeV2-like sequences from different *Formica* host species. The *Formica* virus clade is highlighted in gray and bootstrap values are shown above the branches. The branches between the FeV2-like sequences are preliminary, given that the sequences of these viruses (with ambiguous/uncalled bases) are only partial. An asterisk (*) denotes short FeV2-like sequences.

FeV2-like sequences were found in the RNAseq data from *F. pressilabris, F. fusca, F. cinerea,* and *F. truncorum* (sequences similar over a length of 420 to 9,160 bp, and 79–92% identity; Table 2; Fig. 2), but not from *F. aquilonia* and *F. pratensis*. The FeV2-like viral sequences in *F. pressilabris* and *F. cinerea* were nearly full length, covering 99% and 72%, respectively, of the FeV2 genome, with high identity (over 90%). The FeV2-like sequences from *F. fusca,* and *F. truncorum* were only partially assembled, as good quality sequencing data was unavailable for the transcriptome assembly process. The phylogeny for FeV2 virus contained 23 genomes of the *Iflaviridae* family, and suggest that the FeV2 and FeV2-like fragments comprise a monophyletic taxon with very strong (100%) bootstrap support, and that they cluster with other social insect viruses (deformed wing virus, Kakugo virus, and *Varroa destructor* virus 1) with 100% bootstrap support (Fig. 5; Fig. S1B). Similar to FeV1, additional RNASeq sequencing will be necessary to assess the status of these potential viruses.

The FeV2 virus was found in all castes (males, queens and workers) of *F. exsecta*, but the loads were measurable only in mature workers and queens, and in the pooled male data (Fig. 4A; Table S6). FeV2-like fragments were found in high levels in workers of *F. pressilabris,* intermediate levels in *F. pressilabris* and *F. cinerea* queens, and negligible, in the remaining samples (Fig. 4B; Table S7).

### FeV4 Virus

The third virus, FeV4, contains a linear non-segmented negative sense RNA genome (9866 bp) with a nucleotide composition of 31.16% A, 23.92% U, 22.94% C, 21.98% G (Table 1). The FeV4 genome is monopartite negative strand RNA (Easton & Ling, 2014), and is predicted to contain five ORFs (Fig. 1C), which were annotated as nucleocapsid protein, phosphoprotein, matrix protein, glycoprotein, and viral RNA polymerase, found in other members of *Mononegavirales*. FeV4 blast searches showed similarities (39% amino acid identity, 96% coverage) to the conserved domain mononeg RNA pol (pfam_00946), which is mainly present in *Mononegavirales*, an order of non-segmented negative stand viruses (Easton & Ling, 2014), such as the Midway virus, the Nyamanini nya virus, the Soybean cyst nematode virus, and the Sierra Nevada virus from the *Nyamiviridae* family.

**Figure 6:**
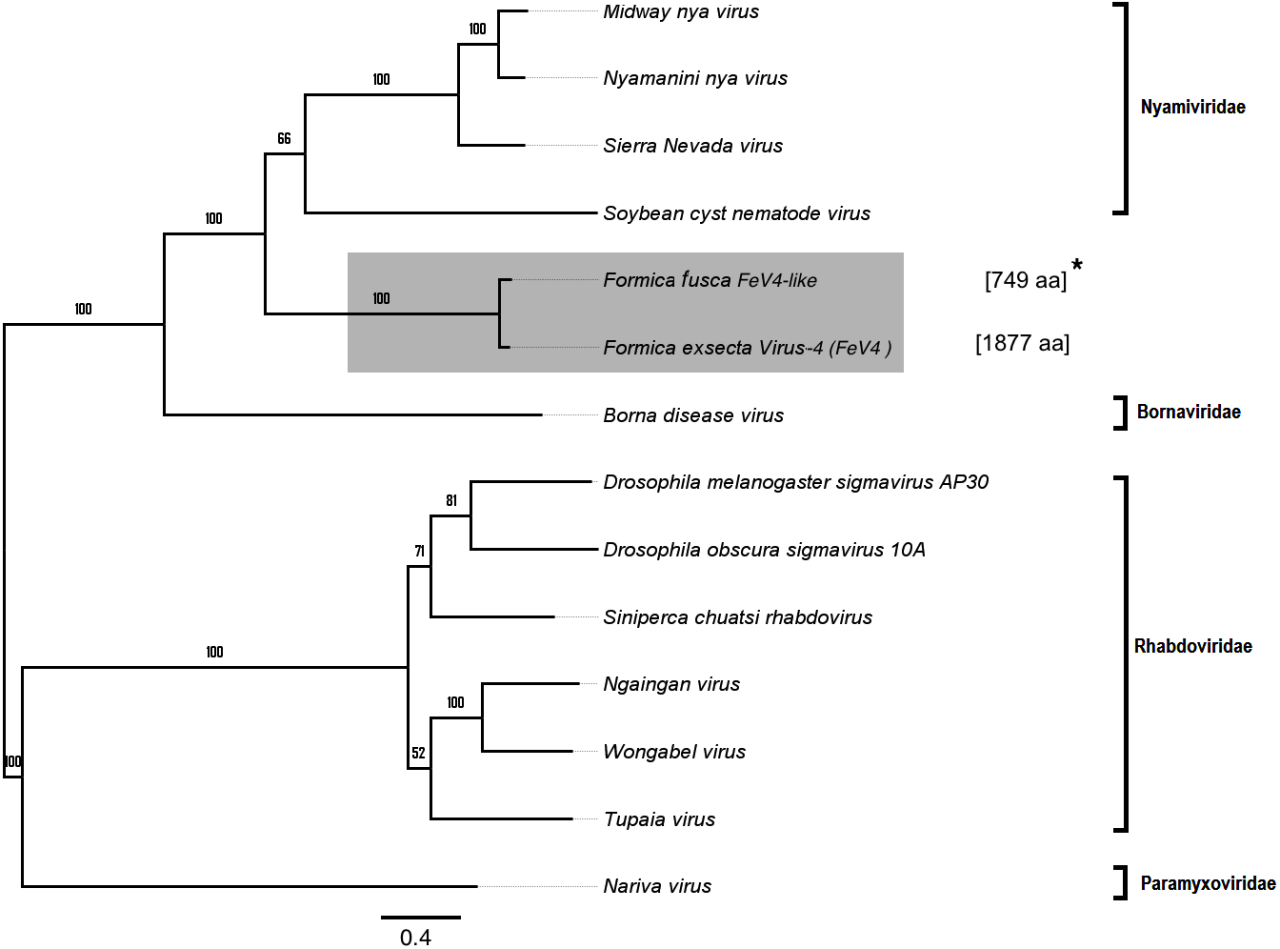
Phylogenetic assignment for FeV4. An unrooted phylogenetic tree was derived from the polymerase protein sequences of the *Mononegavirales* family viruses, which infect insects, along with the partial of the FeV4-like sequences from different *Formica* host species. The *Formica* virus clade is highlighted in gray and bootstrap values are shown above the branches. The branches between the FeV4-like sequences are preliminary, given that the sequences of these viruses (with ambiguous/uncalled bases) are only partial. An asterisk (*) denotes short FeV4-like sequence.

Apart from *F. exsecta,* a fragment with 80% nucleotide identity (87% protein level) to FeV4 was found only in *F. fusca* (Sequence length: 2,280 bp Table 2; Fig. 2), but not in any of the other *Formica* species. The FeV4-like sequence from *F. fusca* clustered together with FEV4 with 100% bootstrap support. For FeV4, the phylogenetic tree revealed several clades, which correspond to different subfamilies present in the order *Mononegavirales* (Fig. 6). The closest relatives of FeV4 belong to Nyamiviridae family with 100% bootstrap support.

Similar to FeV1, FeV4 was also found in all developmental stages of queens and workers of *F. exsecta* (Fig. 4A; Table S6), but showed high levels of viral RNA only in mature queens. Mature workers, as well as immature workers and queens showed intermediate levels of viral RNA, whereas pupae, and the pooled males, showed the lowest levels of viral RNA. The level of FeV4-like sequence was low in workers of *F. fusca*, and no detectable levels were found in the other *Formica* species (Fig. 4B; Table S7).

## Discussion

Using a metatranscriptomic approach, we characterized the complete genomes of two previously identified positive-sense single stranded RNA (+ssRNA) viruses (FeV1, FeV 2), and one new negative-sense single stranded RNA (-ssRNA) virus (FeV4), in the ant *Formica exsecta* in Finland. The three viruses are phylogenetically distinct, and according to our phylogenetic analysis, group into three separate virus families (*Dicistroviridae*, *Iflaviridae*, and *Nyamiviridae*, respectively). They exhibit differences in genome organization; FeV1 is dicistronic, FeV2 is monocistronic, whereas FeV4 is pentacistronic. FeV1 and FeV2 show similarity in sequence and genome organization to many positive-strand RNA viruses that infect ants and honey bees (Chen & Siede, 2007; Valles, 2012; Sébastien et al., 2015). FeV4 is the first negative-sense single stranded RNA virus detected in any species of ant. These viruses were found in all castes, and age classes of *F. exsecta,* and the samples of mature worker ants taken from field colonies show that colonies may be infected by more than one virus. Our results also show that some other ant species of the genus *Formica* contain sequence fragments with close phylogenetic affinity to the three FeV viruses, but these results will need further revalidation.

### Nature of FeV1, FeV2, and FeV4 (viral family)

The overall genomes organization of FeV1 & FeV2 are similar to viruses in the *Dicistroviridae*, *Iflaviridae*, *Picornaviridae*, *Marnaviridae* and *Secoviridae* family. The linear, positive sense, ssRNA genome is 7 to 12.5 kb with a viral genome-linked protein (VPg) covalently linked at the 5′ end and a 3′ polyA tract. The third virus, FeV4, has a genome similar to *Mononegavirales*, but contains unique elements, and may represent a new family of ssRNA viruses. The genome composition of the FeV1 and Hel-Pro-RdRp order, with the di-cistronic structural proteins at the 3′-end of the genome, rather than at the 5′ end, are characteristic to the members of the order Picornavirales (Knowles, 2012). The presence of the intergenic region (IGR) further suggests identity with the *Dicistroviridae* family in the Picornavirales order (Bonning, 2009; Liu, Chen & Bonning, 2015). The closest sequence matches for FeV1 were to the Kashmir bee virus, and these two viruses clustered together on the phylogenetic tree, within the same *Apaviridae* clade that infects social insects. The monopartite structure of FeV2 genome, with non-structural proteins at the 3′ end of the genome, and the presence of all functional domains, suggests affiliation with the *Iflaviridae* family (Chen, Becnel & Valles, 2012; Knowles, 2012; Liu, Chen & Bonning, 2015). The presence of the N-terminal l polypeptide that is characteristic to most iflaviruses, but absent in other members of Picornavirales, the presence of viral coat proteins at the N-terminus, and the non-structural proteins located at the C-terminus in genome (De Miranda & Genersch, 2010; Chen, Becnel & Valles, 2012; Knowles, 2012) further support this conclusion.

We also found a new negative-sense single stranded (-ssRNA) virus (FeV4), with a genome organization consistent with the order *Mononegavirales*. This virus contained two functional domains, Mononeg_RNA_pol and Mononeg_mRNAcap. These domains are present in viruses of the order *Mononegavirales*, including the *Paramyxoviridae* (Easton & Ling, 2014). We found no nucleotide sequence matches in GenBank for FeV4, and only low and partial amino acid sequence identity to the known virus families in *Mononegavirales*. FeV4 clustered with the soybean cyst nematode virus 1, the Nyamanini virus, and the Midway virus (100% bootstrap support), but given that FeV4 differs from these in nucleotide/amino acid sequence identity, and has a UTR/IGR, previously not reported for *Mononegavirales*, it may represent a previously unknown family of -ssRNA viruses.

We found sequence fragments (FeV-like), with similarity to one of the three *Formica exsecta* viruses, from five other ant species of the genus *Formica* (none were found in *F. pratensis,* which may indicate the absence of these viruses in the species, or their incidental absence in this particular sample). Of these, the FeV2-like sequences found in *F. pressilabris* and *F. cinerea* clustered with, were similar in length, and showed high sequence identity to FeV2. Most likely. they represent closely related forms of the FeV2 virus but their status needs to be confirmed. The FeV1-like, and FeV4-like sequences showed reasonable (80–98%) sequence identity with their respective FeV counterparts, but the sequences were short, which precludes firm conclusions. They nonetheless clustered with their respective FeV counterparts in *F. exsecta* with nearly 100% bootstrap support, and the analysis based on short fragments produced a similar topology. Overall, the tree topology of the phylogeny is similar to the one constructed using only complete genome sequences from a previous study (Johansson et al., 2013; Silva et al., 2015; Nouri et al., 2015; Valles et al., 2017a; Valles et al., 2017b). However, to ascertain the exact genetic relationships between the FeV viruses and the FeV-like strains, and to allow assembly of complete genomes for comparison, additional RNA sequencing data is needed (more individuals from different colonies).

Several members of the *Dicistroviridae* have wide host-ranges, and are capable of infecting several insect orders, such as Hymenoptera, Diptera, Hemiptera, Lepidoptera, and Orthoptera (Bonning, 2009). Similarly, many members of the *Iflaviridae* have wide host ranges, including Hymenoptera, Lepidoptera, and Hemiptera (Liu, Chen & Bonning, 2015). Members of the order *Mononegavirales* have an extremely broad range of hosts, and have been discovered in plants, several vertebrate orders, as well as a broad range of insects and other arthropods (Bourhy et al., 2005). Our results show that they are also present in social insects, but the mode of transmission is unclear, and the virus differs in crucial structural properties from those described earlier. Viruses within *Iflaviridae* and the *Aparavirus* clade within *Dicistroviridae* have both vertical and horizontal transmission (Chen et al., 2006; Chen & Siede, 2007), which facilitates dispersal across both life stages and colonies, and paves the way for cross-population, or cross-species infection. Key to cross-transmission are the interactions social insects engage in with members of their own colony, the food resources, the external parasites they have (e.g., mites), and the microbes present in their nest material ( Lindström et al., 2018;  Schmid-Hempel & Schmid-Hempel, 1993). The samples in this study were collected from the same area in Finland, where these species overlap in their distribution and habitat, and may come into contact via shared food sites and temporary social parasitism (Collingwood, 1979; Czechowski, Radchenko & Czechowska, 2002; Seifert, 2007), via exoparasites, such as mites (ants are often covered with these, Sundström, pers. obs.), or via shared cohabitants of their nest mounds ( Lindström et al., 2018; Johansson et al., 2013; Eickwort, 1990;  Schmid-Hempel & Schmid-Hempel, 1993;  Buschinger, 1986; Härkönen & Sorvari, 2018; Elo, Penttinen & Sorvari, 2018). If so, the FeV, and the FeV-like fragments detected in the other *Formica* species may form separate species, or quasispecies (Mordecai et al., 2016). Whether this indeed is the case remains to be determined.

### Differences in infection per caste, and ant species + examples in other species

Of the three FeV viruses, FeV1 had the overall most consistent RNA levels across the tested life stages in *F. exsecta*. Members of the *Aparavirus* clade (*Dicistroviridae*), to which FeV1 belongs, are indeed often expressed in all life stages, even in eggs (Chen & Siede, 2007). FeV1 showed the highest RNA levels in the newly emerged (immature) life stages of *F. exsecta,* echoing the pattern found in SINV-1, which infects *S. invicta* (Hashimoto, Valles & Strong, 2007). In honey bees, these viruses (DWV, KBV, *Varroa destructor* virus-1 etc.) are known to be transmitted by *Varroa* mites (Francis, Nielsen & Kryger, 2013), several species of which have also been associated with *Formica* ants, including *F. exsecta* (Eickwort, 1990; Johansson et al., 2013). Earlier studies suggested that *Varroa* mites transmit the virus to adult bees during feeding (Shen et al., 2005; Locke et al., 2012; Francis, Nielsen & Kryger, 2013). Thus, the increased levels of FeV1 RNA in immature individuals could be mediated via mites present on adult ants. The FeV1-like sequences also showed consistent and measurable levels of RNA in the other *Formica* species, except *F. pratensis*.

The FeV2 virus showed elevated levels of viral RNA in all castes (males, queens, and workers), but only in mature individuals of *F. exsecta,* not in pupae and immature adults. This stands in contrast to honey bees, in which all castes (queens, drones, and workers), and developmental stages (eggs, larvae, pupae, and adults), are infected by *Iflaviridae* viruses (e.g., the DWV virus: honey bees, ants, *Varroa* mites; the Sacbrood bee virus: honey bees; the Kakugo virus: honey bees; the *Varroa destructor* virus-1: honey bees) (Chen et al., 2004; Chen, Higgins & Feldlaufer, 2005; Chen & Siede, 2007; De Miranda & Genersch, 2010; Mordecai et al., 2016). Although FeV2 RNA was not present in detectable levels in pupae, we cannot rule out infections at earlier developmental stages. The variation in viral RNA levels may reflect differences in the ability of different developmental stages or castes to suppress FeV2 infection and/or replication. In particular, the levels of FeV2 RNA were lower in pupae than mature individuals, which may reflect lower infection rates (due to the protective pupal case) and/or multiplication rates (e.g., due to metabolic changes during metamorphosis) in pupae. Elevated levels of FeV2-like sequences were found in four of the six other *Formica* species, without any significant bias according to caste. FeV4 was found in all screened castes and life stages of *F. exsecta*. Mature queens showed higher levels of viral RNA than workers, which may reflect age- or caste-specific variation in prevalence. More extensive sampling is needed to test whether this is indeed the case. In the other *Formica* species, FeV4-like fragments were detected in low amounts only in *F. fusca*.

Viral RNA levels differed considerably between the *F. exsecta* samples obtained by the RNAseq data generated in this study, and the sequence data generated in Morandin et al. (2016). The most likely cause for these differences are the considerable differences in sample sizes, and the number of individuals and colonies pooled for each RNA library. The *Formica* RNASeq data sets generated by Morandin et al. (2016) contained fewer individuals collected from fewer colonies. This highlights the importance of adequate sample sizes and the inclusion of different developmental stages. In the future, the viral loads in different species and castes should be verified with higher number of replicates per species, and sampling targeted to study variation in viral load.

### General

The use of meta-transcriptome data (bulk RNA-Seq) is a powerful approach to characterize viromes, and is key to the recent rapid increase of published virus genomes (Shi et al., 2016). The small sizes of viral genomes often result in high capture rates of viral sequence data, but complications in sequencing data due to low RNA quality and host contamination may affect the completeness of the genome assemblies (Liu, Chen & Bonning, 2015). Our bioinformatic analysis of the three *F. exsecta* viruses (FeV1, FeV2, FeV4) suggest that the genome assemblies of all three viruses belong to the “coding complete” category proposed for virus genome quality standards (Ladner et al., 2014). To fit in this category, there must be a single contig per genome, no gaps, and ORFs must be complete. In addition, NCBI assigned all three genomes as “complete genome” following their own quality criteria. Ultimately, additional characterizations (e.g., Rapid amplification of cDNA ends (RACE) (Olivarius, Plessy & Carninci, 2009; Yeku & Frohman, 2010), further high depth coverage sequencing) would be beneficial to confirm our results, including all non-protein-coding sequences at end (Shi et al., 2016). The FeV-like sequences do not fulfil these requirements, and must therefore be considered preliminary, until more complete sequences are obtained from the host species.

At the point of sampling, no overt disease symptoms were noted in the ants (Johansson et al., 2013). Several close relatives to FeV1 that are found in social insects, such as SINV-1 in *S. invicta* and KBV in the honeybee, commonly persist within brood and adults as non-symptomatic infections (Dall, 1985; Anderson & Gibbs, 1988; Chen & Siede, 2007). However, in both cases disease outbreaks with reduced adult performance and mortality in their wake can occur at different life stages (Valles, 2012). Under environmental stress, SINV-1, KBV and most honey-bee viruses, increase their rate of replication causing overt symptoms, and even death (Allen & Ball, 1996; Chen & Siede, 2007). Close observation of physiology, life span and behavior of infected *F. exsecta* would be required to determine disease symptoms. Disease symptoms may be more visible under infection by FeV2, as its closest relatives, the DWV virus and the Kakugo virus cause wing deformities in emerging honeybees, and changes in behavior and life span, respectively (Bailey, 1971; Chen & Siede, 2007; Dainat et al., 2012; De Miranda & Genersch, 2010; Fujiyuki et al., 2005). In *F. exsecta,* males and queens with deformed wings have been observed from the sampled population (K Dhaygude, pers. obs., 2013), but the underlying cause has not been investigated. To date, no close relatives to FeV4 have been reported from social insects, and in *Mononegavirales* disease symptoms are mostly unknown, with the exception of the Sigma virus that causes vulnerability to CO_2_ exposure in *Drosophila melanogaster* (Possee & King, 2014)*.* We also found cases of co-infection by two FeV strains in *F. exsecta*, cases of which have also been reported in *S. invicta,* and honey bees, where it carries an elevated risk of colony demise (Chen et al., 2004; Allen, Valles & Strong, 2011; Tantillo et al., 2015).

## Conclusions

Here we report a virus (FeV4) which is new to science, with a genetic makeup never described before in any virus found in ants. We further analyzed the prevalence of three viruses (FeV1, FeV2, FeV4) in different life stages and castes of *F. exsecta*, and found differences in virus load between some of these. Our results, along with other recent studies (Shi et al., 2016; Valles, 2012; Valles et al., 2016; Sébastien et al., 2015), demonstrate the use of high throughput sequencing of RNA to detect and identify multiple, and highly diverse RNA viruses. Herein, we also take a step towards elucidating the phylogenetics of these viruses in relation to those that infect other insects. Finally, our study has added important insights in the genomic structure of these viruses. Our findings pave the way towards targeted analyses of host specificity, infection pathways, and host-parasite interactions in social insect hosts, and their viral partners.

##  Supplemental Information

10.7717/peerj.6216/supp-1 Table S1 The number of individuals pooled in each of the totals of 14 libraries sequenced by Institute for Molecular Medicine Finland (FIMM) or Beijing Genomics Institute (BGI) The number of individuals pooled in each of the totals of 14 libraries sequenced by Institute for Molecular Medicine Finland (FIMM) or Beijing Genomics Institute (BGI). Caste and developmental stage of *Formica exsecta* are given together with a number of individuals pooled in each library along with number source colonies in parentheses (Note: Each colony contributed equally in individuals pooling).

10.7717/peerj.6216/supp-2 Table S2 Biosample details of raw sequencing data Biosample details of raw sequencing data. Each biosample includes information on sequencing location, developmental stage, sample name and Sequence read archive (SRA) ID.

10.7717/peerj.6216/supp-3 Table S3 Genes and primer sequences used for RT-PCR verification of viruses Genes and primer sequences used for RT-PCR verification of viruses.

10.7717/peerj.6216/supp-4 Table S4 All virus genome sequences used for the phylogenetic analysis of FeV1, FeV2, and FeV4, with detailed information on virus family, virus name, sequence length (bp), and NCBI ID All virus genome sequences used for the phylogenetic analysis of FeV1, FeV2, and FeV4, with detailed information on virus family, virus name, sequence length (bp), and NCBI ID.

10.7717/peerj.6216/supp-5 Table S5 RT-PCR validation on the original cDNA pools in which the virus was detected, and on 14 colonies collected in July 2013. In 5 colonies no viruses were found, in one colony two viruses were found RT-PCR validation on the original cDNA pools in which the virus was detected, and on 14 colonies collected in July 2013. In 5 colonies no viruses were found, in one colony two viruses were found.

10.7717/peerj.6216/supp-6 Table S6 Raw RPKM and LN-transformed (lnx+1) values (Single individuals level normalization), obtained using either different developmental stages of pooled individuals (queens, workers) or a mixture of these stages (males) of *F. exsecta.* Raw RPKM and LN-transformed (lnx+1) values (Single individuals level normalization), obtained using either different developmental stages of pooled individuals (queens, workers) or a mixture of these stages (males) of *F. exsecta.*

10.7717/peerj.6216/supp-7 Table S7 Raw RPKM and LN-transformed (lnx+1) values (Single individuals level normalization) for seven *Formica* species (Raw data from Morandin et al., 2016) Raw RPKM and LN-transformed (lnx+1) values (Single individuals level normalization) for seven *Formica* species (Raw data from Morandin et al., 2016).

10.7717/peerj.6216/supp-8 Figure S1 Phylogenetic assignment of FeV1 and FeV2 using only short fragment data Phylogenetic assignment of FeV1 and FeV2 using only short fragment data. (A) the FeV1 phylogenetic tree as derived from a 325 bp region similar in length to the FeV1-like sequences, and a few *Discistroviridae* family viruses. (B) the FeV2 phylogenetic tree as derived from a 375 bp region similar in length to FeV2-like sequences and a few *Iflaviridae* family viruses.

## Additional Information and Declarations

### DNA Deposition

The following information was supplied regarding the deposition of DNA sequences: The annotated full genomes of the three viruses are available from GenBank ID: KF500001 (FeV1 virus, 9,554 bp), KF500002 (FeV2 virus, 9,160 bp), MF287670 (FeV4, 9,866 bp). GenBank accession for FeV-like viruses: MF287660–MF287669.

### Data Availability

The following information was supplied regarding data availability: Raw data can be found at NCBI at BioProject: PRJNA213662 and SRP028315.

### New Species Registration

The following information was supplied regarding the registration of a newly described species: GenBank: https://www.ncbi.nlm.nih.gov/nuccore/1444544064
Taxonomy: https://www.ncbi.nlm.nih.gov/Taxonomy/Browser/wwwtax.cgi?id=2306079
This proposed new species [Formica exsecta virus 4] has been submitted to the ICTV for consideration. This name is only valid and official after the ICTV has approved the name, and it has been ratified by the membership.
