# Supplementary material for: Genome organization and molecular characterization of the three Formica exsecta viruses—FeV1, FeV2 and FeV4
Source: PeerJ. 2019 Feb 20;6:e6216. doi: 10.7717/peerj.6216 (PMC6387575; doi:10.7717/peerj.6216)
Supplement: Table S6 — Raw RPKM and LN-transformed (lnx+1) values (Single individuals level normalization), obtained using either different developmental stages of pooled individuals (queens, workers) or a mixture of these stages (males) of F. exsecta. [file peerj-07-6216-s006.docx]

**Table S6. Raw RPKM and LN-transformed (lnx+1) values (Single individuals level normalization), obtained using either different developmental stages of pooled individuals (queens, workers) or a mixture of these stages (males) of *F. exsecta.***

| **Life stage** | **Library** | **Caste** | **FeV1** | |  | **FeV2** | |  | **FeV4** | |
| --- | --- | --- | --- | --- | --- | --- | --- | --- | --- | --- |
|  |  |  | RPKM | LN(RPKM+1) |  | RPKM | LN(RPKM+1) |  | RPKM | LN(RPKM+1) |
|  |  |  |  |  |  |  |  |  |  |  |
| Mixture | Lib-7 | Male | 0.100 | 0.095 |  | 0.035 | 0.0344 |  | 0.233 | 0.209 |
|  |  |  |  |  |  |  |  |  |  |  |
|  |  |  |  |  |  |  |  |  |  |  |
| Pupae | Lib-6 | Worker | 0.123 | 0.116 |  | 0.0 | 0.0 |  | 0.562 | 0.446 |
|  | Lib-3 | Queen | 0.338 | 0.291 |  | 0.0 | 0.0 |  | 0.118 | 0.111 |
|  |  |  |  |  |  |  |  |  |  |  |
|  |  |  |  |  |  |  |  |  |  |  |
| Immature | Lib-5 | Worker | 201.58 | 5.311 |  | 0.0 | 0.0 |  | 2.045 | 1.113 |
|  | Lib-2 | Queen | 49.51 | 3.922 |  | 0.0 | 0.0 |  | 2.537 | 1.263 |
|  |  |  |  |  |  |  |  |  |  |  |
|  |  |  |  |  |  |  |  |  |  |  |
| Mature | Lib-4 | Worker | 0.00041 | 0.00041 |  | 21.946 | 3.1332 |  | 2.988 | 1.383 |
|  | Lib-1 | Queen | 0.00048 | 0.00048 |  | 1.182 | 0.78 |  | 54.56 | 4.018 |
|  |  |  |  |  |  |  |  |  |  |  |
