# Supplementary material for: Genome organization and molecular characterization of the three Formica exsecta viruses—FeV1, FeV2 and FeV4
Source: PeerJ. 2019 Feb 20;6:e6216. doi: 10.7717/peerj.6216 (PMC6387575; doi:10.7717/peerj.6216)
Supplement: Table S7 — Raw RPKM and LN-transformed (lnx+1) values (Single individuals level normalization) for seven Formica species (Raw data from Morandin et al., 2016). [file peerj-07-6216-s007.docx]

**Table S7. Raw RPKM and LN-transformed (lnx+1) values (Single individuals level normalization) for seven *Formica* species (Raw data from Morandin et al 2016).**

| **Species** | **FeV1** |  | |  |  | |  | **FeV2** |  |  |  |  | **FeV4** |  |  |  |  |
| --- | --- | --- | --- | --- | --- | --- | --- | --- | --- | --- | --- | --- | --- | --- | --- | --- | --- |
|  | Queens | | | Workers | | |  | Queens |  | Workers | |  | Queens |  | Workers | | |
|  | RPKM | | LN(RPKM+1) | RPKM | | LN(RPKM+1) | | RPKM | LN(RPKM+1) | RPKM | LN(RPKM+1) |  | RPKM | LN(RPKM+1) | RPKM | LN(RPKM+1) |  |
| *Formica pratensis* | 0 | | 0 | 0 | | 0 |  | 0 | 0 | 0 | 0 |  | 0 | 0 | 0 | 0 |  |
| *Formica aquilonia* | 0.009 | | 0.0088 | 0.008 | | 0.0081 |  | 0 | 0 | 0 | 0 |  | 0 | 0 | 0 | 0 |  |
| *Formica truncorum* | 0.002 | | 0.0016 | 0.012 | | 0.0119 |  | 0.01 | 0.007 | 0 | 0 |  | 0 | 0 | 0 | 0 |  |
| *Formica cinerea* | 0.004 | | 0.0041 | 0.166 | | 0.1533 |  | 4.31 | 1.670 | 0.0009 | 0.001 |  | 0 | 0 | 0 | 0 |  |
| *Formica fusca* | 0.006 | | 0.0061 | 0.201 | | 0.1835 |  | 0 | 0 | 0.074 | 0.071 |  | 0.014 | 0.014 | 0.0040 | 0.004 |  |
| *Formica pressilabris* | 0.001 | | 0.0010 | 0.224 | | 0.2024 |  | 13.14 | 2.649 | 301.20 | 5.711 |  | 0 | 0 | 0 | 0 |  |
| *Formica exsecta* | 0.009 | | 0.0089 | 0.118 | | 0.1114 |  | 414.56 | 6.030 | 0.120 | 0.113 |  | 199.43 | 5.300 | 39.73 | 3.707 |  |
