# Supplementary figures and images for: Genome organization and molecular characterization of the three Formica exsecta viruses—FeV1, FeV2 and FeV4
Source: PeerJ. 2019 Feb 20;6:e6216. doi: 10.7717/peerj.6216 (PMC6387575; doi:10.7717/peerj.6216)

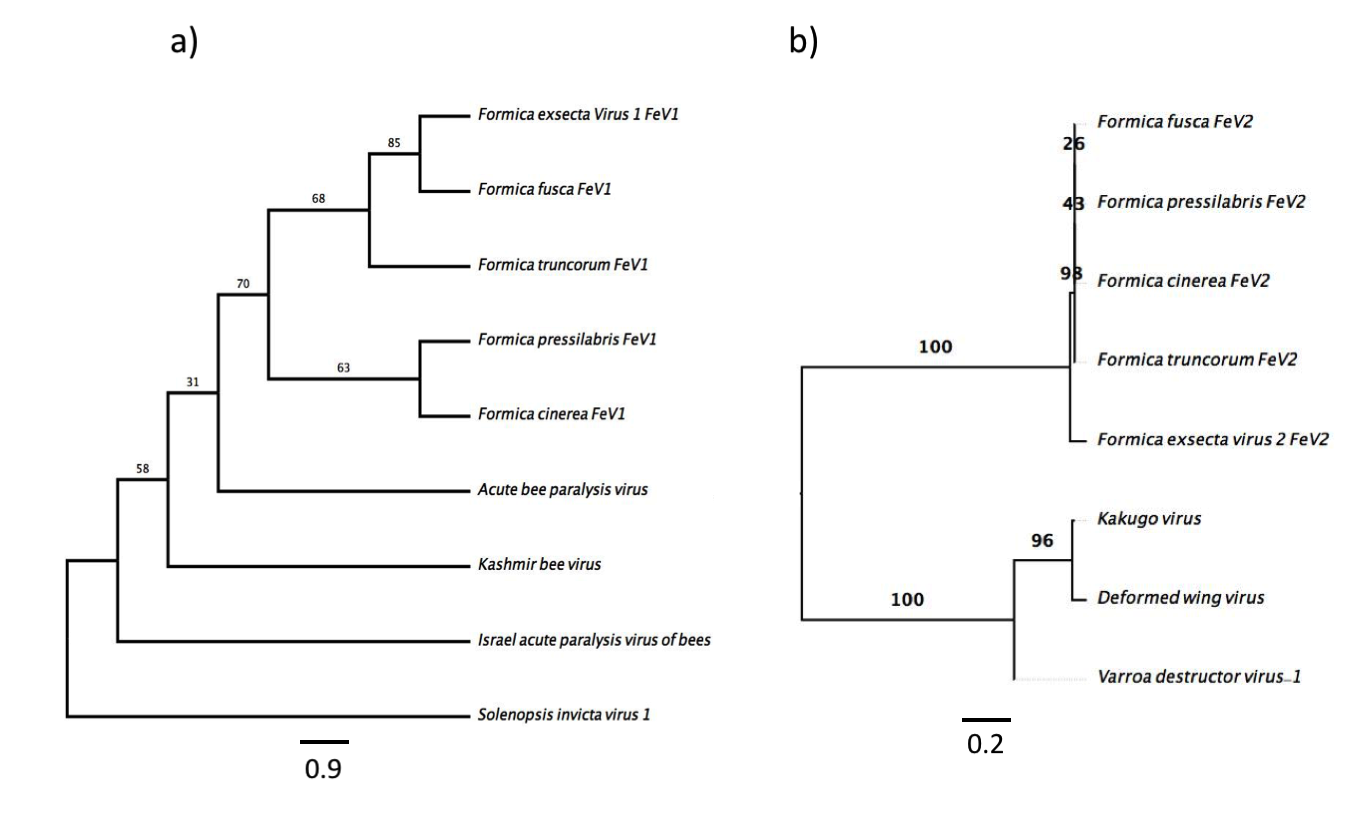

Supplement: Figure S1 — Phylogenetic assignment of FeV1 and FeV2 using only short fragment data. (A) the FeV1 phylogenetic tree as derived from a 325 bp region similar in length to the FeV1-like sequences, and a few Discistroviridae family viruses. (B) the FeV2 phylogenetic tree as derived from a 375 bp region similar in length to FeV2-like sequences and a few Iflaviridae family viruses. [file peerj-07-6216-s008.png]
